# Aging deteriorated perception of urge-to-cough without changing cough reflex threshold to citric acid in female never-smokers

**DOI:** 10.1186/1745-9974-7-3

**Published:** 2011-06-28

**Authors:** Satoru Ebihara, Takae Ebihara, Masashi Kanezaki, Peijun Gui, Miyako Yamasaki, Hiroyuki Arai, Masahiro Kohzuki

**Affiliations:** 1 Department of Internal Medicine and Rehabilitation Science, Tohoku University Graduate School of Medicine, Sendai, Japan; 2 Department of Geriatrics and Gerontology, Institute of Development, Aging and Cancer, Tohoku University, Sendai, Japan

## Abstract

**Background:**

The effect of aging on the cognitive aspect of cough has not been studied yet. The purpose of this study is to investigate the aging effect on the perception of urge-to-cough in healthy individuals.

**Methods:**

Fourteen young, female, healthy never-smokers were recruited via public postings. Twelve elderly female healthy never-smokers were recruited from a nursing home residence. The cough reflex threshold and the urge-to-cough were evaluated by inhalation of citric acid. The cough reflex sensitivities were defined as the lowest concentration of citric acid that elicited two or more coughs (C_2_) and five or more coughs (C_5_). The urge-to-cough was evaluated using a modified the Borg scale.

**Results:**

There was no significant difference in the cough reflex threshold to citric acid between young and elderly subjects. The urge-to-cough scores at the concentration of C_2 _and C_5 _were significantly smaller in the elderly than young subjects. The urge-to-cough log-log slope in elderly subjects (0.73 ± 0.71 point · L/g) was significantly gentler than those of young subjects (1.35 ± 0.53 point · L/g, p < 0.01). There were no significant differences in the urge-to-cough threshold estimated between young and elderly subjects.

**Conclusions:**

The cough reflex threshold did not differ between young and elderly subjects whereas cognition of urge-to-cough was significantly decreased in elderly subjects in female never-smokers. Objective monitoring of cough might be important in the elderly people.

## Background

It has been suggested that the increased incidence of pneumonia with aging may be a consequence of impairment of the cough reflex with senescence [1]. However, the data on cough reflex sensitivity in old age are inconsistent. One study has demonstrated that in elderly people the cough reflex to inhaled ammonia gas is reduced [2]. Another study showed that the cough frequency on inhaling distilled water was significantly lower in elderly subjects than in younger subjects [3]. On the other hand, Katsumata and co-workers measured the cough reflex threshold to citric acid in 110 healthy subjects ranging from 20 to 78 years in age, and found that the cough reflex did not decrease with advanced aging [4].

Aging is attributed to both increasing and decreasing factors for cough reflex sensitivity. Increase in the incidence of cerebrovascular and degenerative neurogenic diseases with aging are strongly associated with impaired cough reflex [5]. Increases in the incidence of gastroesophageal reflux diseases and chronic aspiration with aging are a cause of chronic cough in the elderly [6]. We showed a wide diversity of cough reflex thresholds to citric acid in the elderly nursing home residents [7].

Although the cough reflex is usually referred to as a reflexive defense mechanism mediated at the brainstem level, there is accumulating evidence indicating that human cough is under voluntary control and that higher centers such as the cerebral cortex or subcortical regions have an important role in both initiating and inhibiting reflexive cough [8,9]. Cough is typically preceded by an awareness of an irritating stimulus and is perceived as a need to cough, termed the urge-to-cough [10].

Urge-to-cough is a component of the brain motivation system that mediates the cognitive responses of cough stimuli [11]. The urge is a motivational impulse which relates to how much someone wants something. Studies suggest that the initiation of a reflex cough response is facilitated by the perception of urge-to-cough [12-14]. Heretofore, no study attempted to describe the effect of age on the perception of urge-to-cough.

A lack of motivation that is not attributed to consciousness disturbance, cognitive impairment, or emotional distress, referred as apathy, is one of the most common neuropsychiatric symptoms in the elderly [15], and is reported to increase with age in otherwise healthy community-dwelling individuals [16]. Therefore, it is conceivable to hypothesize that the perception of urge-to-cough is deteriorated in elderly people. The purpose of this study is to investigate the aging effect on the perception of urge-to-cough in healthy individuals.

## Methods

### Subjects

Since gender differences and smoking status differences exist in the cough reflex sensitivity and the perception of urge-to-cough, we focused on female never-smokers in this study [17,18]. Fourteen young and 12 elderly female healthy never-smokers were allocated to evaluate cough related responses to inhaled citric acid. Young healthy female never-smokers were recruited via public postings in and around the Tohoku University School of Medicine campus. Subjects were without history of pulmonary and airway diseases, recent (within 4 weeks) suggestive symptoms, respiratory tract infection and seasonal allergies. Subjects did not take any regular medication.

Elderly female never-smokers were recruited from a nursing home located on the outskirts of Sendai city. We asked all the female residents in the nursing home (41 female residents) and got informed consent from 30 female residents without history of pulmonary and airway diseases, recent (within 4 weeks) suggestive symptoms, respiratory tract infection and seasonal allergies. Of 30 females, 6 subjects with apparent paralysis and history of stroke and Parkinson's disease and syndrome were excluded. Of 24 females, 12 females revealed a difficulty in evaluating the urge-to-cough due to too demented status. Finally, 12 female residents were enrolled for this study. All subjects measured were asked to withhold their tranquilizer use for 36 hours before the study.

The study was approved by the Institutional Review Board of the Tohoku University School of Medicine.

### Cough reflex threshold and urge-to-cough

Cough reflex, urge-to-cough, perception of dyspnea and spirometry were examined at around 2:00 PM for each subject. Simple standard instructions were given to each subject.

Cough reflex threshold to citric acid was evaluated with a tidal breathing nebulized solution delivered by an ultrasonic nebulizer (MU-32, Sharp Co. Ltd., Osaka, Japan) [19]. Citric acid was dissolved in saline, providing a two-fold incremental concentration from 0.7 to 360 mg/ml. The duration of each citric acid inhalation was 1 minute. In the study, cough was defined as a forced expulsive maneuver, usually against a closed glottis, and is associated with a characteristic sound. Based on "cough sound", the number of coughs was counted both audibly and visually by laboratory technicians who were unaware of the clinical details of the patients and the study purpose. Each subject inhaled a control solution of physiological saline followed by a progressively increasing concentration of citric acid. Increasing concentrations were inhaled until five or more coughs were elicited, and each nebulizer application was separated by a 2-min interval. The cough reflex threshold and suprathreshold were estimated by the lowest concentration of citric acid that elicited two or more coughs (C_2_) and the lowest concentration of citric acid that elicited five or more coughs (C_5_) during 1 minute, respectively.

Immediately after the completion of each nebulizer application, the subject made an estimate of the urge-to-cough. The modified Borg scale was used to allow subjects to estimate the urge-to-cough [10]. The scale ranged from "no need to cough" (rated 0) to "maximum urge-to-cough" (rated 10). The urge-to-cough scale was placed in front of the subjects and the subject pointed at the scale number, which was recorded by the experimenter. To assess the intensity of the urge-to-cough, subjects were told to ignore other sensations such as dyspnea, burning, irritation, choking and smoke in the throat. Subjects were told that their sensation of an urge-to-cough could increase, decrease, or stay the same during the citric acid challenges, and that their use of the modified Borg scale should reflect this.

In each subject, the estimated urge-to-cough scores were plotted against the corresponding citric acid concentration using a log-log transformation. Since it is known that there is a linear relationship between estimated urge-to-cough scores and tussive agent concentration on a log-log scale [10,20], the slope and intersection were determined by linear regression analysis on a log-log scale [18]. The thresholds of urge-to-cough in each subject were estimated as an intersection with the X-axis (citric acid concentration axis), indicating the dose of the urge-to-cough score = 1.

### Data analysis

The study protocol was approved by the local ethics committee and informed consent was obtained from all subjects. Data are expressed as mean (SD) except where specified otherwise. The Mann-Whitney *U *test was used to compare between young and elderly subjects. A p value of < 0.05 was considered significant.

## Results

Twenty six subjects who completed the experiments did not experience any side effects. The characteristics of the subjects are summarized in Table 1. Activity of daily living estimated by the Barthel index and cognitive function estimated by MMSE in elderly subjects were significantly lower than those in younger subjects.

**Table 1:** Comparison of characteristics between young and elderly women.

	Young	Elderly	P-value
Number	14	12	
Age (years)	24.6 ± 3.9	85.6 ± 7.1	< 0.0001
Barthel index (scores)	100 ± 0	43.2 ± 22.2	< 0.0001
MMSE (points)	30 ± 0	16.8 ± 8.9	< 0.0001

Data are mean ± S.D. P-value by the Mann-Whitney *U *test. MMSE denotes mini-mental state examination.

**Figure 1:**
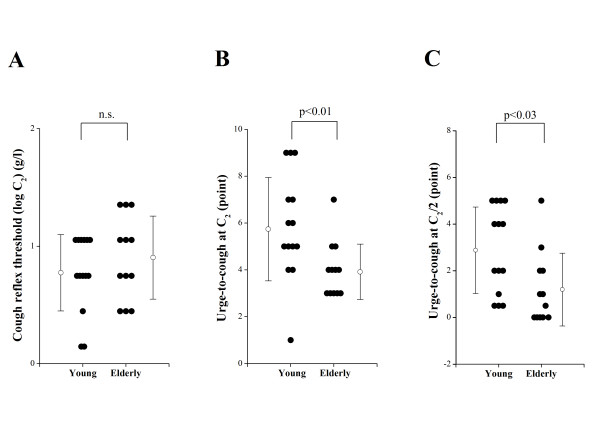
**Comparisons of cough reflex sensitivity and urge-to-cough between young and elderly subjects**. (A) Cough reflex sensitivities expressed as the log transformation of the lowest concentration of citric acid that elicited five or more coughs (C_2_). (B) The urge-to-cough estimated by the Borg scores at C_2 _of each subject. (C) The urge-to-cough estimated by the Borg scores at the concentration of two times dilution of C2 (C_2_/2) of each subject. Closed circles indicate the value of each subject. Open circles and error bars indicate the mean value and the standard deviation in each group, respectively. n.s. denotes not significant.

As shown in Figure 1A, in the cough reflex threshold to citric acid, as expressed by log C_2, _there was no significant difference between young (0.8 ± 0.3 g/l) and elderly subjects (0.9 ± 0.4 g/l). The urge-to-cough scores at the concentration of C_2 _and at the concentration of two times dilution of C_2 _(C_2_/2) were estimated for each subject. The urge-to-cough scores at C_2 _in elderly subjects (4.0 ± 1.2 points) were significantly smaller than those in young subjects (5.9 ± 2.2 points, p < 0.01) (Figure 1B). The urge-to-cough scores at C_2_/2 in elderly subjects (1.2 ± 1.6 points) were also significantly smaller than those in young subjects (2.9 ± 1.9 points, p < 0.03) (Figure 1C).

**Figure 2:**
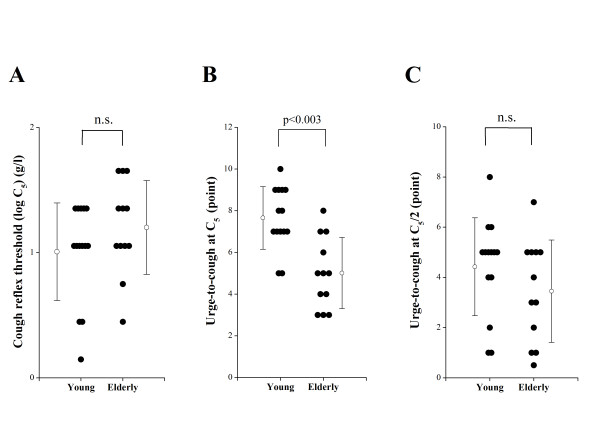
**Comparisons of cough reflex sensitivity and urge-to-cough between young and elderly subjects**. (A) Cough reflex sensitivities expressed as the log transformation of the lowest concentration of citric acid that elicited five or more coughs (C_5_). (B) The urge-to-cough estimated by the Borg scores at C_5 _of each subject. (C) The urge-to-cough estimated by the Borg scores at the concentration of two times dilution of C5 (C_5_/2) of each subject. Closed circles indicate the value of each subject. Open circles and error bars indicate the mean value and the standard deviation in each group, respectively. n.s. denotes not significant.

As shown in Figure 2A, in the cough reflex threshold to citric acid, as expressed by log C_5, _there was no significant difference between young (1.0 ± 0.4 g/l) and elderly subjects (1.2 ± 0.4 g/l). The urge-to-cough scores at the concentration of C_5 _and at the concentration of two times dilution of C_5 _(C_5_/2) were estimated for each subject. The urge-to-cough scores at C_5 _in elderly subjects (5.0 ± 1.7 points) were significantly smaller than those in young subjects (7.6 ± 1.5 points, p < 0.003) (Figure 2B). However, there were no significant differences in the urge-to-cough at C_5_/2 between young (4.4 ± 1.9 points) and elderly subjects (3.5 ± 2.0 points) (Figure 2C).

**Figure 3:**
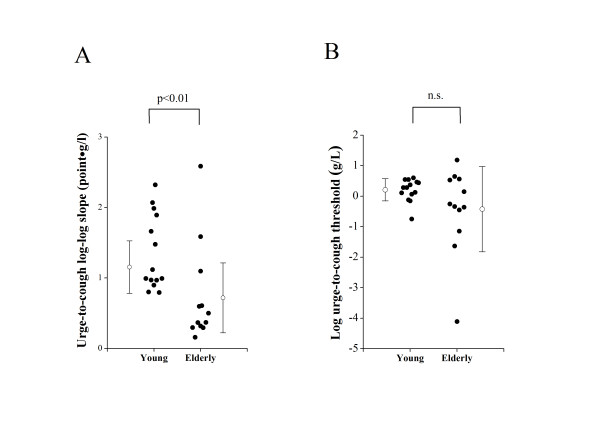
**Comparisons of urge-to-cough between young and elderly subjects**. (A) The urge-to-cough log-log slope by linear regression between log citric acid concentration and the log Borg scores. (B) The urge-to-cough threshold estimated by log citric acid concentration at the log Borg Score of urge-to-cough = 0. Closed circles indicate the value of each subject. Open circles and error bars indicate the mean value and the standard deviation in each group, respectively. n.s. denotes not significant.

The log-log slope between citric acid concentration and the Borg scores of the urge-to-cough were estimated for each subject. As shown in Figure 3A, the urge-to-cough log-log slope in young subjects (1.35 ± 0.53 point · L/g) was significantly steeper than those of elderly subjects (0.73 ± 0.71 point · L/g, p < 0.05). The urge thresholds were estimated as an intersection with the X-axis of the linear regression equation of the log-log relationships between citric acid concentration and the Borg scores of the urge-to-cough. There were no significant differences in the urge-to-cough threshold estimated between young (0.20 ± 0.36 g/L) and elderly subjects (-0.44 ± 1.40 g/L) (Figure 3B), suggesting that an age-related difference in urge-to-cough was raised from the difference in central sensitization process rather than peripheral sensory inputs.

There were no significant relationships between the Barthel index scores and the urge-to-cough log-log slopes among elderly subjects, and between the MMSE scores and the urge-to-cough log-log slopes.

## Discussion

In this study, we showed that cough reflex threshold did not differ between young and elderly subjects whereas the slope for log-log relationship in urge-to-cough intensity as a function of citric acid concentrations was significantly decreased in elderly subjects in female never-smokers.

Our data concerning cough reflex threshold might appear to be inconsistent with previous studies using ammonia gas [2] and distilled water [3]. However, the study using ammonia gas stimuli measured the brief stop in the inspiration which may not necessarily indicate cough. The study using distilled water measured the cough frequency during 30 seconds inhalation. Since causes of the initial cough and the successive cough may differ, these studies may be difficult to compare with our study. It is warranted to study the aging effect on cough reflex threshold using the standard capsaicin method, but such a study has not been performed as far as we know.

Our observation on cough reflex threshold is compatible with Katsumata et al. [4] and is comparable to Fujimura et al. [21] which showed no difference in cough reflex threshold between young and middle-aged females. Aging is associated with both up-regulating and down-regulating factors for cough reflex sensitivities. The gastro-esophageal reflux diseases (GERD), recurrent aspiration, and left ventricular failure, which are common diseases in the elderly, are up-regulating factors of cough [6]. Especially, GERD is the main cause of cough reflex hepersensitivity in the elderly people [7]. On the other hand, the incidence of cerebrovascular and degenerative neurogenic diseases with aging are down-regulating factors for cough [9]. We might not exclude subclinical stages of these diseases. The cough reflex thresholds might be decided by the balance of these factors.

For the first time, we showed that aging inhibits the perception of urge-to-cough without changing the cough reflex threshold. Previous studies showed that decreased perceptions of urge-to-cough in males compared with females [17], current-smokers compared with never-smokers [18], patients with aspiration pneumonia compared with age-matched control [14], and subjects during exercise [22]. Different from aging effect, they are accompanied by significant elevation of cough reflex thresholds. On the other hand, similar with aging, patients with Ondine's curse showed an impaired perception of urge-to-cough despite a normal cough reflex threshold [23]. It is notable that both aged people and patients with Ondine's curse are prone to aspiration pneumonia [24,25], suggesting the importance of urge-to-cough to prevent aspiration pneumonia.

Although cough is usually referred to as a reflex controlled from the brainstem, cough can be also controlled via the higher cortical center and can be related to cortical modulations [6]. Therefore, the depression of cough reflex could be due to the disruption of both the cortical facilitatory pathway for cough and the medullary reflex pathway. Since the urge-to-cough is a brain component of the cough motivation-to-action system [11], depressed urge-to-cough suggests the impairment of motivation and reward pathway for cough, which is located in supra-medulla. Aging is associated with a decline in mental function across multiple domains, including memory and emotional processes [26]. Although it is known that people become more apathic in their normal aging [16], the precise reason has not been elucidated. In addition to the involvement of impaired speed of information processing, attention and executive function, the involvement of brain pathology such as total atrophy and right frontal subcortical circuit pathology have been postulated. Recently, it was reported that deep white matter lesions are associated with apathetic behavior in the elderly [27]. Since the present study has the limitation of lacking brain imaging, we do not know the subclinical brain pathology and its possible association to urge-to-cough in the elderly.

Thus, the observed deterioration in perception of urge-to-cough in the elderly group could be due to aging or a consequence of potentially existing brain disorders. In addition, the study had some limitations due to small sample size. The studies of larger sample size with brain imaging data are warranted.

## Conclusions

The cough reflex threshold did not differ between young and elderly subjects whereas cognition of urge-to-cough was significantly decreased in elderly subjects in female never-smokers. Our study has some clinical implications. Elderly people may not complain of excessive cough because of lack of the cognitive component of cough. Therefore, it might be of importance to monitor cough objectively in order to detect early sign of respiratory infections for elderly people.

## List of abbreviations used

C_2_: the lowest concentration of citric acid that elicited two or more coughs; C_5_: the lowest concentration of citric acid that elicited five or more coughs; GERD: gastro-esophageal reflux diseases.

## Competing interests

The authors declare that they have no competing interests.

## Authors' contributions

SE and TE participated in the design of the study, collected and analyzed data, and drafted the manuscript. KM, PG and MY participated in the design of the study and collected the data. HA and MK participated in design of the study and helped to draft the manuscript. All the authors read and approved the final manuscript.
